# *Candida antarctica* Lipase B Immobilized onto Chitin Conjugated with POSS^®^ Compounds: Useful Tool for Rapeseed Oil Conversion

**DOI:** 10.3390/ijms17091581

**Published:** 2016-09-20

**Authors:** Jakub Zdarta, Marcin Wysokowski, Małgorzata Norman, Agnieszka Kołodziejczak-Radzimska, Dariusz Moszyński, Hieronim Maciejewski, Hermann Ehrlich, Teofil Jesionowski

**Affiliations:** 1 Institute of Chemical Technology and Engineering, Faculty of Chemical Technology, Poznan University of Technology, Berdychowo 4, Poznan 60965, Poland; jakub_zdarta@wp.pl (J.Z.); malgorzata.norman@hotmail.com (M.N.); agnieszka.kolodziejczak-radzimska@put.poznan.pl (A.K.-R.); 2 Institute of Chemical and Environment Engineering, Faculty of Chemical Technology and Engineering, West Pomeranian University of Technology, Pulaskiego 10, Szczecin 70322, Poland; dmoszynski@zut.edu.pl; 3 Faculty of Chemistry, Adam Mickiewicz University in Poznan, Umultowska 89b, Poznan 61614, Poland; maciejm@amu.edu.pl; 4 Poznan Science and Technology Park, Adam Mickiewicz University Foundation, Rubiez 46, Poznan 61612, Poland; 5 Institute of Experimental Physics, Technische Universität Bergakademie Freiberg, Leipziger Str. 23, Freiberg 09599, Germany; hermann.ehrlich@physik.tu-freiberg.de

**Keywords:** enzyme immobilization, chitin, polyhedral oligomeric silsesquioxanes, *Candida antarctica* lipase B, rapeseed oil transesterification

## Abstract

A new method is proposed for the production of a novel chitin-polyhedral oligomeric silsesquioxanes (POSS) enzyme support. Analysis by such techniques as X-ray photoelectron spectroscopy (XPS) and Raman spectroscopy confirmed the effective functionalization of the chitin surface. The resulting hybrid carriers were used in the process of immobilization of the lipase type b from *Candida antarctica* (CALB). Fourier transform infrared spectroscopy (FTIR) confirmed the effective immobilization of the enzyme. The tests of the catalytic activity showed that the resulting support-biocatalyst systems remain hydrolytically active (retention of the hydrolytic activity up to 87% for the chitin + Methacryl POSS^®^ cage mixture (MPOSS) + CALB after 24 h of the immobilization), as well as represents good thermal and operational stability, and retain over 80% of its activity in a wide range of temperatures (30–60 °C) and pH (6–9). Chitin-POSS-lipase systems were used in the transesterification processes of rapeseed oil at various reaction conditions. Produced systems allowed the total conversion of the oil to fatty acid methyl esters (FAME) and glycerol after 24 h of the process at pH 10 and a temperature 40 °C, while the Methacryl POSS^®^ cage mixture (MPOSS) was used as a chitin-modifying agent.

## 1. Introduction

Chitin is a structural aminopolysaccharide found in the cell walls of fungi and diatoms [1], as well as in the exoskeletons of arthropods, like crustaceans [2] and insects [3,4]. Recently, it was shown that it is also possible to isolate chitin from several marine [5] and freshwater sponges [6]. Industrially, however, chitin is obtained mainly from the exoskeletons of shrimps and crabs, which are a seafood processing waste. This waste is an emerging problem in countries where the food industry is based mainly on seafood [7]. Therefore, the development of new methods of chitin utilization is an ongoing trend. The presence of functional groups in the chitin molecule, mainly –OH, as well as –NH_2_ groups, the occurrence of which is a result of a deacetylation process, make this biopolymer suitable for modification [8]. This property, combined with the relatively high thermal [9] and chemical stability of this biopolymer, opens up new areas in which chitin can be utilized, including wastewater treatment [10,11], biomedicine [12], catalysis and biocatalysis [13], production of biosensors [14], extreme biomimetics [15,16,17] etc.

Polyhedral oligomeric silsesquioxanes (POSS) are a specific type of polycyclic compounds with the basic chemical formula (*R*SiO_1.5_)*_n_*, where *n* is an integer and *R* can be a range of organic substituents [18]. Since POSS are usually used as fillers and modifiers of synthetic polymers, the main task of the organic functional groups is to improve the affinity of the inorganic core to the polymer matrix. Recently, a number of papers have been published on the modification of a range of biological polymeric systems by these sophisticated molecules [19,20]. Surprisingly, according to our best knowledge, the modification of chitin with POSS to prepare a new type of support for enzyme immobilization has not previously been reported.

Lipases (EC 3.1.1.3) are the enzymes that are very commonly used in many industrial processes, such as hydrolysis, transesterification, and esterification reactions of a variety of chemical compounds [21]. These enzymes exhibit high catalytic activity in both organic and inorganic solvents, but depending on the reaction environment they can catalyze different chemical transformations [22]. High catalytic activity of the lipases is observed at the organic-aqueous interface. This is directly related to their chemical structure and the construction of their active sites, named interfacial activation [23]. This phenomenon might be explained by the conformational changes occurring in the active center of the peptide under influence of the hydrophobic interface. In these conditions oligopeptide lid of the active site is opening, making it accessible to the reaction substrates [24,25]. Various types of lipases are known from their commercial use in the cosmetic, pharmaceutical, or food industries [26,27,28]. However, during recent years these enzymes have been started to be used in biodiesel production, where they enable reaching high levels of substrate conversion, as well as obtained products at high purity [29,30]. Enzymatically-catalyzed transesterifacion of the vegetable oils with short chain alcohols is a kinetically-controlled process that is influenced by several factors. Among others, the most important is molar ratio of the substrates, temperature, type of the organic solvent and water activity [31]. All of these parameters a correlated with the properties of used lipase and influenced its activity.

Enzyme immobilization involves the attachment of a biocatalyst to a support, producing a catalyst in heterogeneous form [32]. Systems of the immobilized enzymes can be obtained by the various methods, such as adsorption, covalent binding, entrapment, or encapsulation [33,34,35]. The most commonly used is immobilization by adsorption due to its simplicity and low costs. Among many advantages of the immobilization improving and extending activity, selectivity, and resistance of the enzymes to the inhibitors are the most important [36,37,38,39,40]. Furthermore, binding of the biocatalyst to the matrix reduces denaturation and inactivation of the immobilized enzyme in the harsh reaction conditions [41,42,43]. Additionally, multipoint immobilization is particularly important for multimeric forms of the enzymes since it protects this type of the biocatalyst against dissociation and loss of activity [44,45]. However, attachment of the enzyme to the solid carriers may also have an unfavorable effect on the enzyme properties. Creation of the chemical bonds between enzyme particles and a matrix results in the interference in the enzyme structure and its distortion. It may result in inactivation of the biomolecules [46]. Moreover, high enzyme loading and too short a distance between the enzyme and a carrier leads to blocking of the enzyme active sites and creation of the diffusional limitations in the transfer of substrates and products, which trigger the decrease in the biocatalyst activity [47,48,49].

There are many types of support that can be used in this process, and these can be divided into two main groups—organic and inorganic supports [50]. Inorganic supports, such as metal oxides [51], silicas, and minerals, are easily available and offer high stability and resistance to adverse effects of the reaction environment. Nonetheless, they have a relatively small quantity of sites enabling effective attachment of the enzyme, which means that the protein molecules are quickly washed off the support surface [52].

A very wide range of organic matrices may be used in the immobilization process, including many polymers [53,54]. An interesting alternative, providing completely new possibilities of application, is the use of biopolymer supports of organic origin. Notable among these are cellulose, chitin, chitosan [55,56,57,58,59], chitin-chitosan, and chitin-lignin hybrids [60]. Such matrices, in view of their natural origin, are not only biocompatible, but are also non-toxic, which extends the potential scope of their use. Additionally, the presence in their structure of reactive functional groups not only facilitates attachment of the enzyme, but also makes the support surface much more susceptible to modification, for example using glutaraldehyde [61,62,63]. An additional advantage of using chitin as a support is the variety of forms in which it can be used. Depending on requirements, it can be used in the form of powder, flakes, beads, nanoscale whiskers, fibers [64], or 3D-scaffolds [65].

At present, a very rapidly-developing area in which enzymes immobilized on biopolymer matrices may be used is the production of biosensors. Substances with chitin as support may be used in the production of biodetectors for the detection of nucleic acids, or as immunosensors [14].

The application of the lipases in many various processes, as catalysts, has been reported previously in a large number of scientific publications [66,67,68]. The use of the natural polymers, such as chitin, chitosan, or cellulose are known as stable and reusable supports for enzyme immobilization as it was reported earlier by many researchers [69,70,71]. Therefore, in the present work, innovative research was undertaken to carry out a process of modification of industrial chitin of crustacean origin using compounds from the group of polyhedral oligomeric silsesquioxanes (POSS compounds), and then to use the resulting hybrid materials as matrices in the process of immobilizing lipase from the yeast *Candida antarctica*. Use of the polyhedral oligomeric silsesquioxanes increase affinity of the matrix to the enzymes particles by increasing its hydrophobicity. Additionally, use of this type of modifying agent can help reduce diffusional resistance associated with the transport of substrates and products by increasing the distance between enzyme particles and the surface of the matrix. Moreover the presence of the numerous of functional groups in the structure of silsesquioxane compounds facilitate attachment of the various groups of enzymes.

Analysis by such methods as X-ray photoelectron spectroscopy (XPS) and ^13^C cross-polarization magic angle spinning nuclear magnetic resonance (^13^C CP MAS NMR) indirectly confirmed functionalization of chitin surface by POSS compounds. Tests of the hydrolytic activity of the chitin-POSS-enzyme systems show with strong evidence the retention of catalytic ability by the immobilized biocatalysts. Moreover, obtained products are characterized by good thermal, chemical, as well as operational, stability. In this study we also report utilization of the produced catalytic systems as an efficient and environmentally friendly biocatalysts in the transesterification of rapeseed oil. Products of this reaction (fatty acid methyl esters (FAME) and glycerol) may find their potential applications in many branches of industry, such as petrochemical industry or as substrates for the synthesis of a wide range of chemical compounds.

## 2. Results

### 2.1. Chitin Surface Functionalization

#### 2.1.1. Surface Analysis

The surface composition of the pure chitin and the chitin-POSS hybrid materials was examined by means of X-ray photoelectron spectroscopy (XPS). The elemental composition calculated from the XPS data is given in Table 1.

Considering the chemical formula of chitin to be (C_8_H_13_O_5_N)*_n_*, and disregarding hydrogen atoms, which are not detected by XPS analysis, the chemical composition of chitin is given as 57 at% carbon, 35 at% oxygen, and 7 at% nitrogen.

The surface of all chitin-POSS hybrids contains a significant concentration of silicon atoms, ranging from 15 to 19 at% depending on the type of POSS modifier. The high concentration of silicon on the surface of the modified chitin confirms that the POSS compounds were successfully attached to the surface of the chitin support.

**Figure 1:**
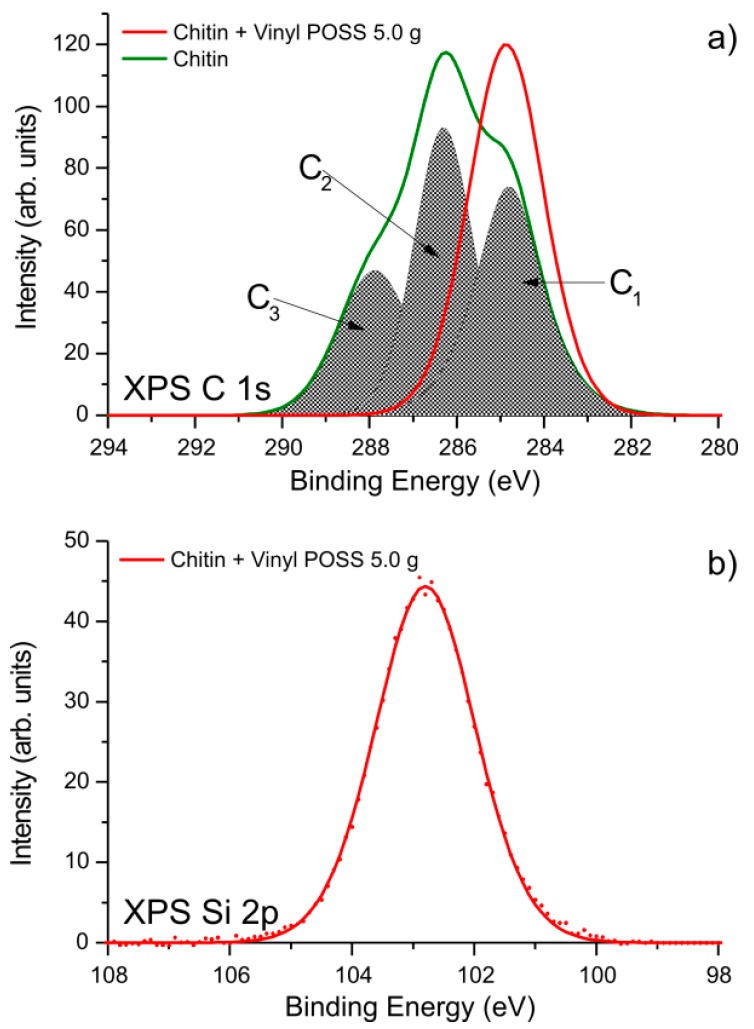
(**a**) C 1s spectrum for chitin and chitin-vinyl POSS hybrid; (**b**) Si 2p spectrum for chitin-vinyl POSS hybrid.

Detailed XPS spectra were used to examine the chemical state of the substances composing the surface of the studied materials. In Figure 1a the C 1s peaks obtained from pure chitin and a hybrid sample composed of α-chitin and Vinyl POSS^®^ cage mixture (VPOSS) are compared. C 1s peaks were examined for all studied hybrid samples. Since they barely differ from one another, only one example is shown in Figure 1a for simplicity. The XPS C 1s peak of chitin has a maximum at a binding energy of 286.4 eV. This position was set arbitrarily due to the procedure used for the correction of charge effects. Carbon atoms in vinyl POSS are bound only to other carbon atoms, hydrogen, and silicon; therefore, the maximum of the C 1s peak for this compound is expected at a binding energy of 285.0 eV. The correct alignment between the component C_1_ attributed to non-functionalized carbon atoms in the chitin structure, and the maximum of the C 1s spectrum obtained for the chitin-VPOSS hybrid, confirms the correct charge compensation. The component C_2_ comprises signals originating from the functional groups C–O–C, C–OH, and C–N–C. The carbon denoted by the asterisk in the *C–O–C=O group may also contribute to that signal. The high energy component C_3_, at a binding energy of about 288.0 eV, corresponds to the set of functional groups C=O, O–C–O and N–C=O. The shape of the C 1s peak for chitin resembles the spectra reported for modified chitin structures [71,72]. The C 1s line for the hybrid chitin-VPOSS sample is a symmetric peak at binding energy 285.0 eV.

As mentioned previously, it is attributed to sp^2^ and sp^3^ carbon atoms, bonded either with a second carbon or with hydrogen or silicon atoms. This justifies the relatively high full width at half maximum (FWHM) of this peak (~2.0 eV). Comparison of the spectra obtained from chitin and the chitin-VPOSS hybrid sample indicates that on the surface of the latter sample the chitin structure is not observed, the support being covered completely by the POSS compound.

In Figure 1b the XPS Si 2p spectrum is shown for the chitin-VPOSS sample. XPS Si 2p lines observed for other POSS-modified samples were virtually identical, and for simplicity they are not shown. The XPS Si 2p line is symmetric, with a maximum at a binding energy of 102.7 eV.

#### 2.1.2. Raman Spectroscopy Results

**Figure 2:**
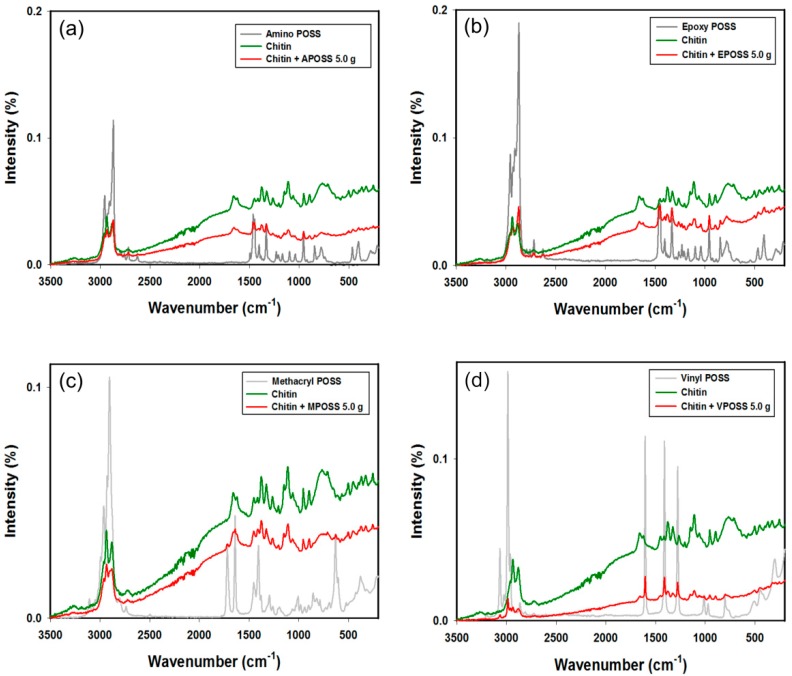
Raman spectra of α-chitin, POSS compounds and the resulting hybrid materials: (**a**) chitin-APOSS; (**b**) chitin-EPOSS; (**c**) chitin-MPOSS; and (**d**) chitin-VPOSS.

The Raman spectra of α-chitin, Epoxycyclohexylisobutyl POSS^®^ (EPOSS), Aminoethylaminopropylisobutyl POSS^®^ (APOSS), Methacryl POSS^®^ cage mixture (MPOSS), Vinyl POSS^®^ cage mixture (VPOSS), and selected chitin-POSS^®^ hybrid materials are presented in Figure 2.

Analysis of the Raman spectra of the chitin-POSS hybrid materials reveals the presence of signals both from the support and the POSS compounds. Moreover, the bands’ intensity changed as a consequence of overlapping of the bands characteristic for chitin and oligomeric polysilsesquioxanes. The band at 403 and 463 cm^−1^ in the EPOSS and APOSS spectra are assigned to the Si–O–Si out-of-plane bending mode, the band at 843 cm^−1^ to the C–Si deformation mode, and the band at 1038 cm^−1^ to the Si–O–Si stretching mode (from the POSS cage). Several vibrations can be assigned to the various side chains of the silsesquioxanes: the band at 1230 cm^−1^ to the CH_2_ wagging deformation, the band at 1460 cm^−1^ to the CH_3_ rocking deformation, and the band at 2718 cm^−1^ to the CH stretching mode.

There is also a notable change in the intensity of particular signals that occur both in chitin and in the POSS compounds. The chitin used has a signal at 2945 cm^−1^ with a greater intensity than that at 2883 cm^−1^. Following functionalization of the surface with POSS compounds the situation is reversed, and the signal at the lower wavenumber value, as a result of effective surface modification, becomes stronger. An analogous situation is found in the case of the signals at 1384 and 1329 cm^−1^.

In the vinyl POSS spectrum, –C=CH_2_ stretching vibrations (from the vinyl group) at 1605 cm^−1^ and CH stretching vibrations (characteristic for vinyl compounds) at around 2990 cm^−1^ are observed, as reported by Boscher et al. and Jalvaro et al. [73,74].

The spectra of the products also feature a signal at 1959 cm^−1^, which can be attributed to CH_2_, CH_3_, or Si–CH_3_ stretching vibrations [75]; this provides additional evidence of the presence of the POSS compounds.

Additional analysis, confirming effective chitin modification, such as scanning electron microscope (SEM), Fourier transform infrared spectroscopy (FTIR) as well as ^13^C cross-polarization magic angle spinning nuclear magnetic resonance (^13^C CP MAS NMR) were performed (Figures S1–S3 and Table S1).

#### 2.1.3. Thermogravimetric Analysis of the Chitin-POSS Materials

**Figure 3:**
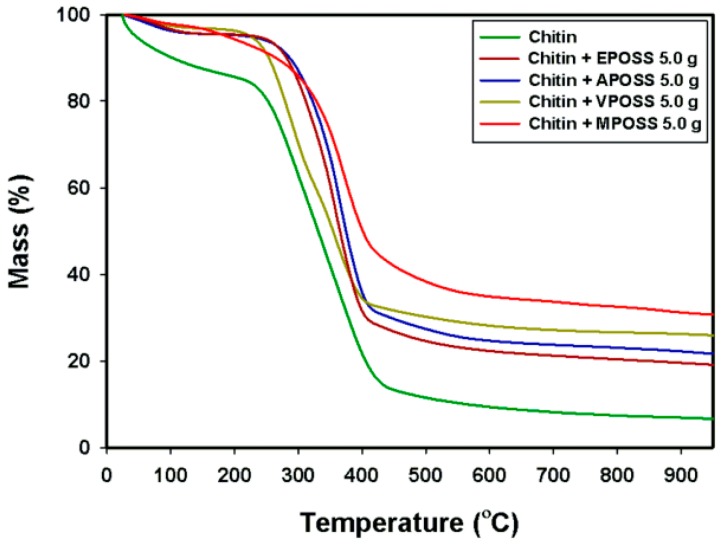
Thermogravimetric (TG) curves for α-chitin and for the products obtained by functionalization with various POSS compounds.

Chitin offers relatively high thermal stability, as can be seen in Figure 3. The curves show two phases of mass loss: the first in a temperature range of 20–150 °C (ca. 15% mass loss), ascribed to the loss of water which is physically adsorbed and/or weakly hydrogen-bonded to chitin molecules [76,77], and the second in a range of 250–450 °C (ca. 60% mass loss), related to the thermal degradation of the polymer chain [78,79].

### 2.2. Characterization of Products Following Lipase Immobilization

#### 2.2.1. Fourier Transform Infrared Spectroscopy (FTIR) Analysis of the Products Following Immobilization

**Figure 4:**
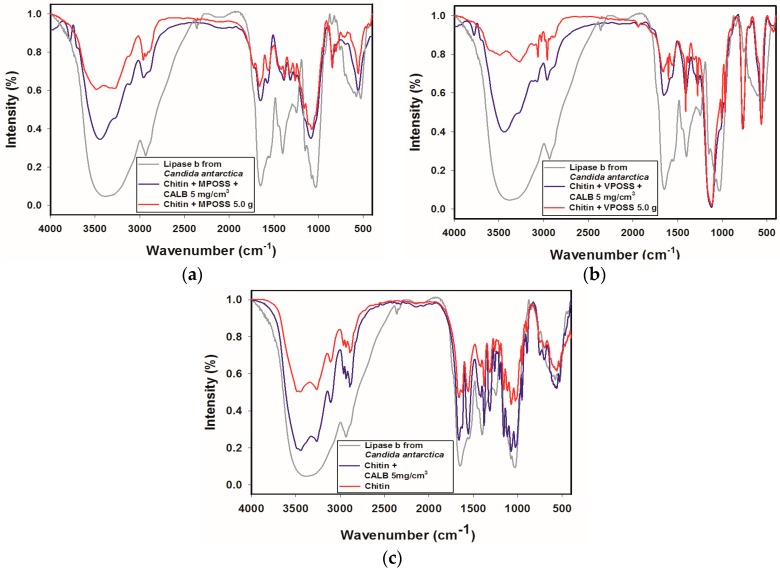
Results of FTIR analysis of the native lipase B from *Candida antarctica* (CALB), chitin, chitin-POSS hybrid materials, and products after enzyme immobilization: (**a**) chitin + MPOSS + CALB; (**b**) chitin + VPOSS + CALB; and (**c**) chitin + CALB.

To get insight into the immobilization of the lipase onto the modified and unmodified chitin surface, Fourier transform infrared spectroscopy (FTIR) was used. The FTIR spectra obtained for the lipase B from *Candida antarctica*, the α-chitin matrix modified by various POSS compounds and the products following immobilization are presented in the Figure 4.

The spectrum of the enzyme contains signals at wavenumber 2937 cm^−1^, which correspond to C–H stretching vibrations in CH_2_ and CH_3_ groups, as well as a wide, intense band with a maximum at 3476 cm^−1^ assigned to stretching vibrations of –OH groups, and a band of N–H stretching vibrations, masked by the former. Particular attention should be drawn to the signals at 1641, 1538, and 1256 cm^−^^1^ that are linked to the presence of amide I, amide II, and amide III bonds, respectively [80]. Below 1000 cm^−1^ there are several signals assigned to C–C bonds.

#### 2.2.2. Retention of Hydrolytic Activity of Products after Immobilization

The retention of the hydrolytic activity of the products following immobilization was evaluated spectrophotometrically and obtained results are presented in Table 2. The initial concentration of the lipase solution used in enzyme immobilization was 5 mg/cm^3^. The process of immobilization was carried out for 1, 24, and 96 h on chitin and chitin functionalized with 5 g of the octasubstituted POSS compounds (MPOSS and VPOSS).

Among others, the factors that influence on the retention of the catalytic activity of the obtained products are immobilization time and the type of surface modifier used. The immobilized onto unmodified chitin CALB exhibit the highest retention activity after 1 h of the process (71.4%). Meanwhile, lipase immobilized onto chitin modified by VPOSS and MPOSS showed the best catalytic properties after 24 h of the process; 81.4% and 87.l%, respectively.

#### 2.2.3. Evaluation of the Stability of the Products after Immobilization

**Figure 5:**
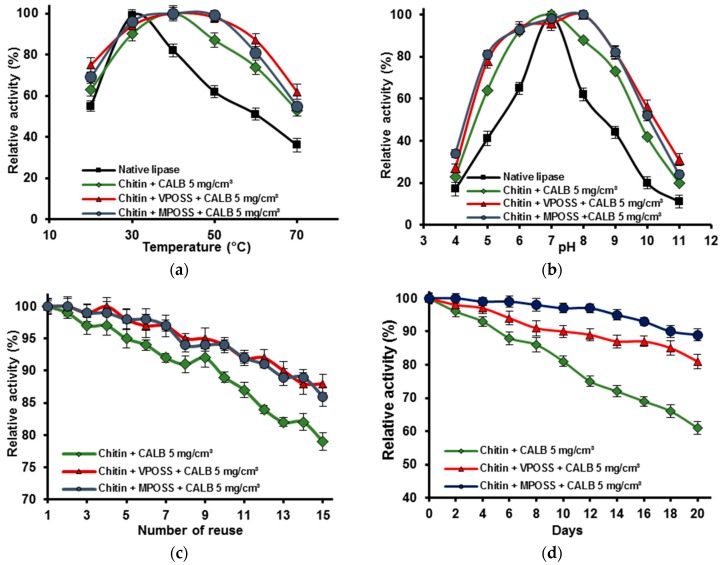
(**a**) Thermal stability; (**b**) effect of the pH; (**c**) reusability; and (**d**) storage stability of the immobilized lipase B from *Candida antarctica*.

Effect of pH and temperature, as well as preservation of high activity and stability during storage and catalysing subsequent reaction cycles are crucial parameters determining potential applications of the immobilized lipase. Stability of the products obtained after 1 (unmodified chitin) or 24 h (modified chitin) of the immobilization from the solution at concentration of 5 mg/cm^3^, calculated based on spectrophotometric measurements, are presented in Figure 5.

The effect of the temperature on the activity of free and immobilized lipase onto unmodified and modified chitin is presented in Figure 5a. Native enzyme exhibit its maximum activity at 30 °C, while for all products after immobilization, it is shifted to 40 °C. When the temperature increases, activity of the free enzyme rapidly decreases, and at 70 °C reached less than 40% of its initial value. Lipase immobilized onto chitin modified by POSS compounds, possesses much higher thermal stability and at 70 °C retains about 60% of initial activity.

The activity of the free and immobilized lipase at various pH values is presented in Figure 5b. Results show that pH has a significant impact on the activity of both free and immobilized enzyme. Native and immobilized onto unmodified chitin lipase exhibit maximal activity at pH 7. Meanwhile, optimal pH of immobilized enzyme onto modified by POSS chitin is shifted into more a basic region (pH 8). It is worth pointing out that free enzyme retains about 60% of initial activity in a narrow pH range (6–8), while immobilized lipase possesses about 80% of its original activity in a pH range from 6–9.

For practical applications it is important that lipase after immobilization not only retain catalytic properties but also preserve high catalytic activity after many reaction cycles, as well as during storage. As it can be seen in Figure 5c, lipase immobilized onto the chitin surface exhibits 79% of its initial activity after 15 operations, while enzymes attached to the biopolymer matrix modified MPOSS and VPOSS retain 89% and 87%, respectively, after the same amount of reuse.

The changes in the activity of the immobilized lipase during storage at 4 °C over 20 days were also evaluated and results are presented in Figure 5d. CALB immobilized onto MPOSS-chitin retain over 90% of initial activity, while the bioactivity of the enzyme onto VPOSS-chitin material slightly decrease and reached 85%. Retention of catalytic activity of chitin + CALB is lower, but still at high level (over 60%) in comparison with native lipase, that after 15 days retain less than 10% of its initial activity.

#### 2.2.4. Rapeseed Oil Transesterification with Immobilized CALB

The methanolysis of rapeseed oil has been selected as a trial reaction to verify the catalytic properties of the obtained systems following immobilization. The process mentioned above is a useful tool for production of a novel biofuels [45], as well as allows obtaining glycerol as a byproduct [46]. The effect of various reaction conditions, such as pH, temperature, process duration, and various biocatalytic systems properties was investigated to evaluate the best reaction parameters. For all of these experiments systems characterized by the highest catalytic activity retention were selected.

Table 3 presents results of the methanolysis reaction under 40 °C with an oil:methanol volume ratio 5:1 at various pH and process times.

The activity of the immobilized CALB increases when pH of the reaction environment increases and reached a maximum at pH 10, regardless of the used modifying agent. Total conversion of the triglycerides is observed at pH 10 after 24 h of the process with the use of the chitin-MPOSS-CALB biocatalytic system. After this process the mixture contains over 90% of the FAME and about 87% of the theoretical amount of glycerol. Application of the other catalytic products makes it impossible to achieve total oil transformation but still allows over 80% (chitin-CALB) and 90% (chitin-VPOSS-CALB) conversion of the triglycerides.

Table 4 shows effect of the temperature and process duration on the catalytic activity on the immobilized CALB during methanolysis reaction.

The conversion of the rapeseed oil increases when temperature increases, and reached its maximum at 40 °C after 24 h of the reaction, regardless of the used catalytic system. The total conversion of the vegetable oil is possible with the use of the lipase immobilized onto chitin modified by MPOSS. It is worth mentioning that a significant decrease of the conversion value is observed at temperatures of 50 and 60 °C.

Table 5 summarises the results of the rapeseed oil transesterification, in different process duration, at optimal reaction conditions.

Conversion of the transesterification increases with prolongation of the reaction time, as it is expected. 24 h of the process duration was selected as the optimum time of this process. After this time total transformation of the triglycerides into FAME and glycerol is noticed, when chitin-MPOSS-CALB was used as the biocatalyst. Although 72 h of the process duration allows to achieve total conversion of the rapeseed oil within the use of all biocatalytic systems, but this period of time is economically unfavourable.

## 3. Discussion

### 3.1. Chitin Surface Functionalization

#### 3.1.1. XPS Analysis

The observed values of the surface composition of the chitin material are relatively close to the theoretical values, confirming the viability of XPS analysis as a tool to examine the quantitative composition of the considered materials. The oxygen-to-carbon ratio for chitin is almost 0.5, as reported previously [65].

Several different silicon atom environments are possible in organosilicon compounds. They are usually described by capital letter symbols: M for mono [(CH_3_)_3_SiO_1/2_], D for di [(CH_3_)_2_SiO_2/2_], T for tri [(CH_3_)SiO_3/2_], and Q for quaternary [SiO_4/2_], to represent siloxy units by indicating the number of oxygen atoms attached to the silicon [74]. The maximum of the XPS Si 2p line (Figure 1b) is typically attributed to the T-type environment of silicon atoms [74,81]. It indicates that no transformation of the POSS cage has taken place upon modification of chitin.

#### 3.1.2. Raman Spectroscopy

The α-chitin and POSS spectra exhibit signals characteristic for the functional groups contained in those compounds [5,75,82,83,84,85]. Detailed data on the main signals confirming the modification of chitin with POSS compounds are presented in the Supplementary Materials (Table S2). Moreover, it should be emphasized, that spectra of the chitin-VPOSS hybrid materials exhibit the greatest changes relative to the spectrum of the matrix. A probable factor behind this is the type and quantity of silsesquioxane substituents. Vinyl POSS has eight substituents, compared with the monosubstituted APOSS and EPOSS.

#### 3.1.3. Thermal Stability Analysis

The observed increase in the onset degradation temperature for all modified samples indicates that chitin-POSS hybrids have higher thermal stability than pure α-chitin. The reduced rate of mass loss and higher char values for all modified samples indicate that POSS compounds deposited on the chitin surface inhibit the single-step reaction of thermal depolymerization of the chitin molecular structure [17] and dehydration of the polysaccharide rings in the hybrids [65]. A similar phenomenon has been observed for polyurethanes by Lewicki et al. [18].

### 3.2. Characterization of Products Following Lipase Immobilization

As an innovative supports for lipase immobilization, octasubstituted POSS compounds (MPOSS and VPOSS) were selected due to the presence of a number of functional groups in the structure of this type of POSS. Moreover, functionalization of the chitin surface by this compounds increases the affinity of the support to the enzyme particles. Selected products after lipase immobilization were taken under investigation to confirm effective enzyme immobilization and evaluation of the catalytic activity and stability of these systems. Obtained results, presented and commented below, proved that CALB was successfully immobilized and remains active in the catalytic tests.

#### 3.2.1. FTIR Analysis

The FTIR spectrum of the lipase features many characteristic signals for the enzyme, which have maxima at similar wavenumber values to bands appearing on the modified chitin spectrum. This is due to the similarity of the functional groups making up chitin and those contained in the structure of the enzyme. Note should be taken, however, on the intensification of the band originating from hydroxyl and amine groups in the products following immobilization, as well as the shift in its maximum towards a higher wavenumber value, which suggests the formation of hydrogen bonds between the protein and the modified support. Similar changes in the FTIR spectra have been noted by Svendsen [86] and additional analysis confirmed that this is due to hydrogen bonds created between support and the enzyme. There are also shifts towards lower wavenumber values (respectively 1634 and 1531 cm^−1^) in the maxima of the signals for amide I and II bonds. Shifting of these bands was also reported by Dousseau and Pezolet [87] and indicates the retention of the catalytic activity of the products. Moreover, according to Wong et al., this observation is a proof of the effective attachment of the biocatalyst to the matrix [88].

#### 3.2.2. Retention of Hydrolytic Activity of Products after Immobilization

Retention of the highest catalytic activity by the immobilized lipase after 24 h of the process was also reported in our previous work, related to immobilization of the lipase [89]. Moreover, it should be noted that use of the polyhedral silsesquioxanes allows increased retention of the hydrolytic activity, in comparison with unmodified chitin.

Higher retention of hydrolytic activity by the lipase immobilized onto modified chitin can be explained by the presence of numerous of functional groups in the structure of POSS compounds. This fact allows attaching a greater amount of the enzyme, increasing hydrolytic activity. Additionally, use of the POSS compounds helps to maintain a longer distance between enzyme particles and between enzyme particles and the surface of the chitin, reducing enzyme diffusional limitations and, as a consequence, keep the catalytic activity at a high level. These observations are confirmed by the results obtained by Ching-Ching et al. [90] which claimed that length of surface modifying agent have significant impact on the properties of the immobilized enzymes. The highest retention of activity by the lipase immobilized on chitin-MPOSS material can be explained by the presence of carbonyl and vinyl bonds in the structure of this silsesquioxane that allow to create stable interactions between the enzyme and a support and longer alkyl chains that reduce steric constraints.

#### 3.2.3. Effect of the Various Reaction Conditions on the Stability of the Products after Immobilization

The research by Gomes et al. conducted with lipase immobilized onto functionalized chitin indicated its optimum at temperature 45 °C and at pH 7.5 [91]. These data corresponds with the results already mentioned in this publication. However, in comparison with this study, CALB immobilized onto chitin modified by POSS compounds is characterized by the wider pH and temperature range, which exhibits over 80% of its initial activity.

Based on the obtained results, it is clear that immobilization enhanced the stability of the lipase B from *Candida antarctica*. That might be explained by the creation of strong interactions between the lipase and chitin matrix, which reduces the occurrence of conformational changes into the enzyme structure caused by the heat and drastic pH as reported by Saylan et al. [92]. Additionally, these interactions enhance rigidity and stabilize the entire structure of the enzyme, protecting them by the denaturation process [93]. It should also be noticed that strong interactions between the enzyme and a matrix might decrease leaching of the enzyme from the chitin surface [94]. Moreover, according to Narwal et al., lipase attached to the chitin surface is protecting against inactivation caused by the negative effect of the reaction components, particularly solvents [95].

#### 3.2.4. Rapeseed Oil Transesterification with Immobilized CALB

Findings related to the optimal time of the process (24 h) correlate with the observations made by Caballero et al. that indicate 24 h as the optimal duration of this reaction [96]. Obtained results proved that lipase immobilized onto modified chitin retain good catalytic activity even under alkaline conditions. Moreover, it should be emphasizing that addition of the NaOH (up to 150 μL at pH 10 in this study) helps to achieve high total conversion values but never acts as a catalyst of these reactions, as it was reported earlier [97,98]. Conversion of rapeseed oil with methanol, without immobilized lipase, even at pH 10, after 24 h of the process, does not exceed 15%.

The most effective surface-modifying agent considered is MPOSS, which enables immobilization of the significant amount of the lipase, as well as reduce diffusional limitations. Thus, total conversion of the triglycerides is observed. Meanwhile the enzyme attached to the unmodified chitin or chitin functionalized by VPOSS, at optimal conditions, allows achieving conversion at levels of 83% and 92%, respectively. The presented results are in agreement with the results of the hydrolytic activity. A decrease of the conversion value is observed at temperature 50 and 60 °C, which is connected with the partial thermal inactivation of the immobilized enzyme at higher temperatures as it is reported by Luna et al. (2013) [99]. Presented results prove high catalytic properties of this system and are in contradiction with the results presented by Babaki et al. [100]. Applied by these authors, lipase B from *Candida antarctica* immobilized onto Santa Barbara Amorphous (SBA) silica allows only partial conversion of the oil to FAME.

Presented results clearly show that products following immobilization may fulfil an important role as efficient biocatalysts in the transesterification of rapeseed oil with methanol. Use of the lipase immobilized onto the chitin surface functionalized by Methacryl POSS cage mixture enabled total conversion of the triglycerides under mild conditions (pH 10, T = 40 °C) after 24 h of the process, generating the mixture containing 90% of the theoretical amount of FAMEs and 10% of the theoretical amount the glycerol. Furthermore, the foregoing results confirmed findings made based on the hydrolysis of p-NPP into p-NP, related to the highest hydrolytic activity, as well as good chemical and thermal stability of the chitin-MPOSS-CALB biocatalytic systems (Section 2.2.2 and Section 2.2.3).

#### 3.2.5. Suggested Mechanism of the Chitin Modification and Enzyme Immobilization

From the point of view of the stability of the resulting systems it is important to determine the nature of the interactions between molecules and define a mechanisms of chitin functionalization and enzyme immobilization processes.

The character of the functional groups presented in the side chains of octasubstituted POSS (MPOSS and VPOSS), the high stability of the acetamid bonds, as well as carrying out the processes in the mild conditions, without catalysts, caused the formation of the new chemical bonds between chitin and POSS or between POSS and enzyme particles is hampered.

**Figure 6:**
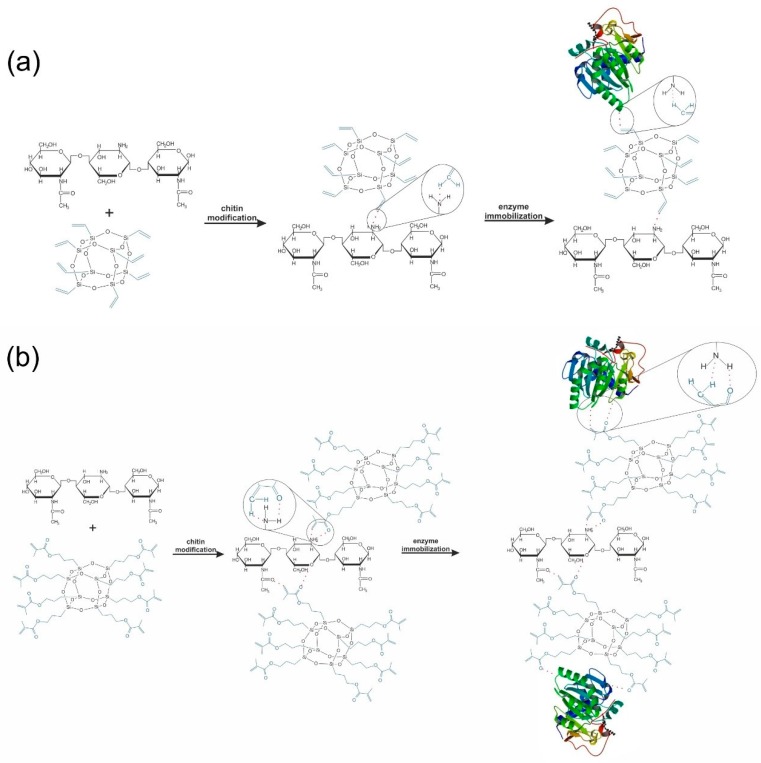
Proposed mechanism for the formation of: (**a**) chitin + VPOSS + CALB; and (**b**) chitin + MPOSS + CALB systems. Hydrogen bonds are marked by red dotted line.

Partial deacetylation of the chitin generates the presence of the –NH_2_ groups in its structure. This groups show an affinity to the functional groups contained in the POSS compounds. This observation, coupled with the results of the XPS and FTIR analyses, may suggest that interactions between the biopolymer surface and silsesquioxanes compounds are probably based on hydrogen bonds. The same groups in modifying agent particles (C=O and C=C) and –NH_2_ groups in the enzyme structure are involved in the creation of interactions during the immobilization process. Slight shifts in maximal wavenumbers of selected bands in the FTIR spectra before and after immobilization might imply that the mechanism of lipase binding to the modified chitin is mostly based on hydrogen bonds. Interactions formed in both processes, in detail, are presented in Figure 6.

Moreover, the hydrophobic character of the chitin surface after its modification by POSS compounds increases the affinity of the matrix to the enzyme. This may be explained by the lipase phenomena called interfacial activation, resulting in its tendency to be adsorbed on the hydrophobic surfaces [101,102,103,104].

## 4. Materials and Methods

### 4.1. Reagents

α-Chitin from crab shells, lipase type B from *Candida antarctica* (CALB), 4-nitrophenyl palmitate (p-NPP), gum arabic and Triton X-100 were obtained from Sigma-Aldrich (Saint Louis, MO, USA). Methacryl POSS^®^ cage mixture (MPOSS), Aminoethylaminopropylisobutyl POSS^®^ (APOSS), Epoxycyclohexylisobutyl POSS^®^ (EPOSS), and Vinyl POSS^®^ cage mixture (VPOSS) were purchased from Hybrid Plastics (Hattiesburg, MS, USA). Propan-2-ol and NaOH was purchased from Chempur (Gliwice, Poland), and 10 mM phosphate buffer at pH 7 was obtained from Amresco Company (Solon, OH, USA). Commercially available rapeseed oil was bought from a local factory.

### 4.2. Functionalization of the Chitin Surface

Commercial chitin was ground in a mechanical mortar, and then was classified using a metallic sieve of mesh diameter 80 μm. The support prepared in this way was introduced in a quantity of 10 g into a round-bottomed flask, and 30 cm^3^ of a solution of the modifier (5.0 g of a selected POSS in an appropriate solvent) was added. The system was mixed for 1 h at room temperature at a rate of 800 rpm. After that time, the solvent was evaporated using a vacuum evaporator, and the remaining material was dried at 100 °C.

### 4.3. Immobilization of Lipase onto the Modified Chitin Matrix

The process of immobilization of lipase B from *Candida antarctica* on both modified and unmodified support involved placing 500 mg of the support in a conical flask and adding a 15 cm^3^ solution of the enzyme, at concentration of 5 mg/cm^3^, in a phosphate buffer at pH 7. The mixture was shaken using a shaking device for a specified time (1, 24 and 96 h) at ambient temperature. The samples were then filtered under reduced pressure and dried at room temperature for 48 h.

### 4.4. Experimental Techniques

X-ray photoelectron spectra were obtained using Al Kα (hν = 1486.6 eV) radiation with a Prevac system equipped with a Scienta SES 2002 electron energy analyzer (Scienta Omicron, Taunusstein, Germany) operating at constant transmission energy (Ep = 50 eV). The spectrometer was calibrated using the following photoemission lines (with reference to the Fermi level): EB Cu 2p_3/2_ = 932.8 eV, EB Ag 3d_5/2_ = 368.3 eV and EB Au 4f_7/2_ = 84.0 eV. The instrumental resolution, as evaluated by the full-width at half maximum (FWHM) of the Ag 3d_5/2_ peak, was 1.0 eV. The samples were loosely placed in a grooved molybdenum sample holder. The analysis chamber during experiments was evacuated to better than 1 × 10^−9^ mbar. Charging effects were corrected using the C 1s peak ascribed to the aliphatic carbon bindings (CH_x_) and set to 285.0 eV. In the case of the chitin sample the maximum of the C 1s peak was set to 286.4 eV, in accordance with Wysokowski et al. [11]. The XPS lines of the other observed elements were shifted correspondingly. The reproducibility of the peak positions thus obtained was ±0.1 eV. The surface composition of the samples was obtained on the basis of the peak area intensities of the C 1s, O 1s, Si 2p and N 1s transitions using the sensitivity factor approach and assuming homogeneous composition of the surface layer.

The Raman scattering spectra were investigated within a spectral range of 3500–250 cm^−1^. The non-polarized Raman spectra were recorded in a back scattering geometry, using the inVia Renishaw micro-Raman system (New Mills, UK). The inVia Raman spectrometer allowed the recording of Raman spectra with a spatial resolution of about 1 μm. The spectral resolution was 4 cm^−1^. A laser operating at 520 nm was used as excitation light.

Thermogravimetric analysis were performed using a Jupiter STA 449F3 apparatus (Netzsch, Germany). Measurements were carried out under nitrogen flow (10 cm^3^/min) at a heating rate of 10 °C/min, over the temperature range 25–950 °C, with an initial sample weight of approximately 5 mg.

Fourier transform infrared spectroscopy (FTIR) was carried out using a Bruker Vertex 70 spectrophotometer (Billerica, MA, USA). The materials were analyzed in the form of tablets, made by placing a mixture of anhydrous KBr (ca. 250 mg) and 1.5 mg of the tested substance in a steel ring under a pressure of 10 MPa. The tests were performed at a resolution of 0.5 cm^−1^ in the wavenumber range 4000–400 cm^−1^.

### 4.5. Evaluation of Hydrolytic Activity of Lipase after Immobilization

The hydrolytic activity of the immobilized lipase was determined according to previously described procedures using p-NPP as a substrate [60]. For the hydrolysis reaction two solutions (S1 and S2) were prepared and mixed. S1 consist of 0.038 g of p-NPP dissolved in 10 cm^3^ of propan-2-ol. S2 consist 0.1 g of the gum arabic and 0.4 g of the Triton X-100 in 90 cm^3^ of the phosphate buffer at pH 7. All reactions were carried out for 2 min at 30 °C with stirring at 1000 rpm. For this reaction 5 mg of the free enzyme and appropriate amount of the product after immobilization consisting 5 mg of the lipase was used. The spectrophotometric measurement were made at wavelength 410 nm. Activity retention (*A_R_*) after immobilization was calculated according to the Equation (1):

(1) $$\;A_{R}=\frac{A_{I}}{A_{0}}\;\times 100\%$$

where *A_I_* denotes the activity of the immobilized lipase, *A*_0_ denotes the initial activity of the lipase. For the evaluation of the following products stability and reusability, systems characterized by the highest catalytic activity retention were selected.

#### 4.5.1. Products after Immobilization Stability

Thermal stability was determined in the temperature range of 20–70 °C. Effect of pH on the free and immobilized CALB was examined in the pH range 4–11 by measurement of immobilized CALB catalytic activity. All of these parameters have been determined based on the hydrolysis reaction of p-NPP carried out for 2 min with stirring at 1000 rpm. For all samples, the effect of the pH was evaluated at the temperature of 40 °C, while the effect of the temperature was evaluated at pH 7.

#### 4.5.2. Storage Stability and Reusability

Experiment was conducted to compare the stability of CALB immobilized on various carriers. Immobilized lipase was stored at 4 °C in phosphate buffer (pH 7) for 20 days and every two days catalytic activity was tested. The activity of each sample was expressed as a percentage of its residual activity compared to the initial activity. The reusability of the immobilized lipase was evaluated spectrophotometrically, based on the hydrolysis of p-NPP over 15 following catalytic cycles.

### 4.6. Methanolysis Reaction of the Rapeseed Oil

The conversion of the rapeseed oil systems characterized by the highest catalytic activity retention were and the process was conducted as follows. To 100 cm^3^ glass flasks, an appropriate amount of the product following immobilization containing 10 mg of the immobilized lipase and 10 cm^3^ of the rapeseed oil and methanol in volume ratio 5:1 were added. The process was performed at a temperature range of 20–60 °C and pH range 6–10 for 12 and 24 h. The appropriate pH environments were reached by adding various quantities of the sodium hydroxide.

FAME and glycerol quantification analysis were conducted according to modified method described by Luna et al. [99]. The PEGASUS 4D GCxGC-TOFMS gas chromatograph (LECO Corp., Joseph, MI, USA) connected to a BPX5 (5% phenyl equivalent) capillary column (SGE Int., Melbourne, Australia) were used. The ion source and transfer line temperature were set at 250 °C. Helium was used as carrier gas, with a flow of 1.0 cm^3^/min. After splitless injection, the oven temperature was set and maintained 5 min at 55 °C, then it has been applied a heating ramp to 300 °C at a rate of 10 °C/min and maintaining the temperature of the oven at for 15 min. Hexadecane was used as an internal standard.

### 4.7. Statistical Analysis

All measurements were made in triplicate and analyzes statistically using SigmaPlot 11 software (Systat Software Inc., San Jose, CA, USA).

## 5. Conclusions

In summary, a process has been carried out whereby the surface of a chitin matrix was functionalized with compounds from the group of polyhedral oligomeric silsesquioxanes (POSS). Results of the performed analysis confirm the effectiveness of the proposed method of modification of chitin. The materials obtained were then used as matrices in the process of *Candida antarctica* lipase B immobilization. Results of FTIR analysis indirectly confirm the effective immobilization of the enzyme on the surface of an appropriately prepared matrix. Hydrolytic activity tests showed that lipase after immobilization onto chitin modified by POSS exhibited higher retention of catalytic activity than enzyme attached to the unmodified carrier. The undertaken analysis proved also that this systems can be characterized by high thermal and pH stability, as well as retained catalytic activity during storage and catalyzing following reaction cycles. Produced chitin-POSS-CALB systems were used as an efficient biocatalysts in transesterification of the rapeseed oil, where they can play a role of the green catalysts. Obtained results proved that total conversion of the triglycerides was achieved. Moreover, immobilized lipase exhibit good catalytic activity even at higher pH, which is not commonly observed in lipases. During the study, the effect of various reaction conditions on the conversion of the rapeseed oil was also investigated. It should be emphasizing that produced matrices (chitin-POSS systems) are also suitable for the other groups of the enzymes, but further investigations in this research area have to be undertaken.

## Figures and Tables

**Table 1:** Elemental composition of the surface of the obtained chitin-polyhedral oligomeric silsesquioxanes (POSS) hybrid materials calculated by XPS analysis.

Sample Name	Surface Composition (at%)			
	C	O	Si	N
Chitin	62.9	30.8	–	6.2
Chitin + Vinyl POSS 5.0 g	60.7	21.1	16.6	1.6
Chitin + Methacryl POSS 5.0 g	51.7	29.0	18.8	0.4
Chitin + Amino POSS 5.0 g	57.2	23.5	17.3	2.0
Chitin + Epoxy POSS 5.0 g	60.5	23.7	14.9	0.9

**Table 2:** Retention of the hydrolytic activity of products after immobilization.

Sample Name	Immobilization Time (h)	Retention of Hydrolytic Activity (%)
Chitin + CALB 5 mg/cm^3^	1	71.4 ± 2.4
	24	68.5 ± 2.3
	96	63.2 ± 2.6
Chitin + VPOSS + CALB 5 mg/cm^3^	1	74.8 ± 2.1
	24	81.4 ± 2.2
	96	75.8 ± 2.1
Chitin + MPOSS + CALB 5 mg/cm^3^	1	78.2 ± 2.4
	24	87.6 ± 2.3
	96	73.4 ± 2.0

**Table 3:** Effect of the pH and reaction time on the chemical composition, * relative content of FAME (%), ** relative content of glycerol (% *w*/*w*), and *** conversion (%) after methanolysis of rapeseed oil. As the relative content, the measured mass of the substance to theoretical mass of the substance after total methanolysis is considered. Reaction conditions: 7.5 cm^3^ rapeseed oil, 1.5 cm^3^ methanol, 10 mg of the immobilized lipase, T = 40 °C.

pH	Time (h)	Chitin + CALB			Chitin + VPOSS + CALB			Chitin + MPOSS + CALB		
		*	**	***	*	**	***	*	**	***
6	12	7.5 ± 0.1	2.2 ± 0.1	7 ± 0.1	9.7 ± 0.2	2.2 ± 0.1	9 ± 0.1	14.0 ± 0.1	3.2 ± 0.1	13 ± 0.2
	24	11.8 ± 0.1	3.2 ± 0.1	11 ± 0.2	14.0 ± 0.2	3.2 ± 0.1	13 ± 0.2	20.4 ± 0.3	5.4 ± 0.2	19 ± 0.3
7	12	13.9 ± 0.2	4.3 ± 0.1	13 ± 0.1	10.7 ± 0.2	3.2 ± 0.1	10 ± 0.2	16.2 ± 0.2	3.2 ± 0.1	15 ± 0.2
	24	20.4 ± 0.2	5.4 ± 0.2	19 ± 0.2	19.3 ± 0.3	5.4 ± 0.1	18 ± 0.4	24.7 ± 0.3	6.5 ± 0.1	23 ± 0.3
8	12	34.6 ± 0.3	49.5 ± 0.2	36 ± 0.5	46.2 ± 0.5	54.8 ± 0.3	47 ± 0.5	51.9 ± 0.5	63.4 ± 0.3	54 ± 0.5
	24	45.0 ± 0.5	55.9 ± 0.3	46 ± 0.7	58.2 ± 0.5	66.7 ± 0.3	59 ± 0.7	70.6 ± 0.6	75.3 ± 0.4	71 ± 0.7
9	12	48.2 ± 0.5	57.0 ± 0.3	49 ± 0.6	63.7 ± 0.7	66.7 ± 0.4	64 ± 1.0	62.4 ± 0.6	68.8 ± 0.3	63 ± 0.6
	24	67.7 ± 0.7	71.0 ± 0.3	68 ± 0.9	76.7 ± 0.8	79.6 ± 0.5	77 ± 1.2	88.5 ± 0.8	88.2 ± 0.5	89 ± 1.2
10	12	53.0 ± 0.6	63.4 ± 0.2	54 ± 0.7	69.0 ± 0.7	68.8 ± 0.3	69 ± 0.8	72.5 ± 0.7	77.4 ± 0.3	73 ± 0.8
	24	82.6 ± 0.7	87.1 ± 0.3	83 ± 1.0	91.6 ± 0.8	91.4 ± 0.4	92 ± 1.4	99.4 ± 1.1	98.9 ± 0.6	98 ± 1.8

**Table 4:** Effect of the temperature and reaction time on the chemical composition, * relative content of FAME (%), ** relative content of glycerol (% *w*/*w*), and *** conversion (%) after methanolysis of rapeseed oil. As the relative content, the measured mass of the substance to theoretical mass of the substance after total methanolysis is considered. Reaction conditions: 7.5 cm^3^ rapeseed oil, 1.5 cm^3^ methanol, 10 mg of the immobilized lipase, pH 10.

T (°C)	Time (h)	Chitin + CALB			Chitin + VPOSS + CALB			Chitin + MPOSS + CALB		
		*	**	***	*	**	***	*	**	***
20	12	21.7 ± 0.2	3.2 ± 0.1	20 ± 0.3	27.7 ± 0.3	9.7 ± 0.1	26 ± 0.4	30.7 ± 0.3	34.4 ± 0.2	31 ± 0.4
	24	42.0 ± 0.4	52.7 ± 0.2	43 ± 0.6	53.4 ± 0.7	60.2 ± 0.3	54 ± 0.6	57.0 ± 0.7	67.7 ± 0.2	58 ± 0.8
30	12	51.6 ± 0.4	55.9 ± 0.2	52 ± 0.5	60.5 ± 0.7	65.6 ± 0.2	61 ± 0.5	65.6 ± 0.8	69.9 ± 0.2	66 ± 0.7
	24	65.7 ± 0.6	68.8 ± 0.1	66 ± 0.6	80.4 ± 0.7	87.1 ± 0.3	81 ± 0.8	86.5 ± 0.8	91.4 ± 0.4	87 ± 0.9
40	12	53.0 ± 0.6	63.4 ± 0.2	54 ± 0.7	69.0 ± 0.7	68.8 ± 0.3	69 ± 0.8	72.5 ± 0.7	77.4 ± 0.3	73 ± 0.8
	24	82.6 ± 0.7	87.1 ± 0.3	83 ± 1.0	91.6 ± 0.8	95.7 ± 0.4	92 ± 1.4	99.4 ± 1.1	98.9 ± 0.6	98 ± 1.8
50	12	47.5 ± 0.5	52.7 ± 0.2	48 ± 0.7	62.4 ± 0.9	68.8 ± 0.2	63 ± 0.7	74.3 ± 0.6	81.7 ± 0.4	75 ± 0.7
	24	69.5 ± 0.7	75.3 ± 0.2	70 ± 0.9	83.8 ± 1.2	86.0 ± 0.3	84 ± 1.1	90.5 ± 1.1	95.7 ± 0.4	91 ± 1.4
60	12	68.6 ± 0.6	73.1 ± 0.2	69 ± 0.8	72.5 ± 0.6	77.4 ± 0.2	73 ± 1.0	82.6 ± 0.7	87.1 ± 0.3	83 ± 0.9
	24	72.4 ± 0.8	89.2 ± 0.2	74 ± 1.1	77.6 ± 0.8	88.2 ± 0.4	79 ± 1.2	88.1 ± 1.6	90.3 ± 0.7	87 ± 2.8

**Table 5:** Effect of the reaction time on the chemical composition, * relative content of FAME (%), ** relative content of glycerol (% *w*/*w*), and *** conversion (%) after methanolysis of rapeseed oil. As the relative content, the measured mass of the substance to theoretical mass of the substance after total methanolysis is considered. Reaction conditions: 7.5 cm^3^ rapeseed oil, 1.5 cm^3^ methanol, 10 mg of the immobilized lipase, pH 10, T = 40 °C.

Time (h)	Chitin + CALB			Chitin + VPOSS + CALB			Chitin + MPOSS + CALB		
	*	**	***	*	**	***	*	**	***
1	3.3 ± 0.1	0.0 ± 0.1	3 ± 0.1	7.6 ± 0.2	1.1 ± 0.1	7 ± 0.2	12.0 ± 0.2	1.1 ± 0.1	11 ± 0.2
2	10.9 ± 0.1	1.1 ± 0.1	10 ± 0.2	16.3 ± 0.2	2.2 ± 0.1	15 ± 0.2	20.7 ± 0.2	2.2 ± 0.1	19 ± 0.2
6	31.0 ± 0.3	31.2 ± 0.2	31 ± 0.4	45.4 ± 0.5	51.6 ± 0.3	46 ± 0.5	51.7 ± 0.4	54.8 ± 0.2	52 ± 0.6
12	53.0 ± 0.6	63.4 ± 0.2	54 ± 0.7	69.0 ± 0.7	68.8 ± 0.3	69 ± 0.8	72.5 ± 0.7	77.4 ± 0.3	73 ± 0.8
24	82.6 ± 0.7	87.1 ± 0.3	83 ± 1.0	91.6 ± 0.8	95.7 ± 0.4	92 ± 1.4	99.4 ± 1.1	98.9 ± 0.6	98 ± 1.8
72	99.9 ± 1.4	96.8 ± 0.5	98 ± 2.3	99.8 ± 1.6	98.9 ± 0.6	98 ± 2.1	99.7 ± 1.2	97.8 ± 0.7	98 ± 1.9

## Supplementary Materials

Supplementary materials can be found at www.mdpi.com/1422-0067/17/9/1581/s1.
