# Supplementary material for: Candida antarctica Lipase B Immobilized onto Chitin Conjugated with POSS® Compounds: Useful Tool for Rapeseed Oil Conversion
Source: Int J Mol Sci. 2016 Sep 20;17(9):1581. doi: 10.3390/ijms17091581 (PMC5037846; doi:10.3390/ijms17091581)
Supplement: Supplementary file 1 [file ijms-17-01581-s001.pdf]

Supplementary Materials: *Candida antarctica* Lipase B Immobilized onto Chitin Conjugated with POSS® Compounds: Useful Tool for Rapeseed Oil Conversion

Jakub Zdarta, Marcin Wysokowski, Małgorzata Norman, Agnieszka Kołodziejczak-Radzimska, Dariusz Moszyński, Hieronim Maciejewski, Hermann Ehrlich and Teofil Jesionowski

1. Experimental Section

Experimental Techniques

The morphology of the chitin-POSS materials was examined on the basis of scanning electron microscopy SEM images recorded from an EVO40 scanning electron microscope (Zeiss, Oberkohen,, Germany). Before testing, the sample was coated with Au for a time of 30 seconds using a Balzers PV205P coater (Oerlikon Balzers Coating, Brugg, Switzerland).

To monitor the course of the synthesis and to verify the products' structure, spectra from ^{13}C CP MAS NMR (75 MHz) were recorded on a Varian XL 300 (Varian, Palo Alto, CA, USA) spectrometer at room temperature. All chemicals were used as obtained from suppliers without any further purification.

2. Results and Discussion

2.1. Chitin Surface Functionalization

2.1.1. Morphological Characterization

Figure S1 shows SEM photos of α-chitin before (a) and after modification with various types of polyhedral oligomeric silsesquioxanes (b–e).

Chitin has a heterogeneous morphological structure, and its surface exhibits grooves and irregular particles of variable shape and size [1]. However, the images of POSS-modified chitin (b–e) reveal small structures with regular shapes, their presence being a result of the effective modification of the chitin surface.

Analysis of the images and of the quantities of geometrical shapes appearing on them indicates that the type of POSS used is significant, because each of the compounds produces a different coverage of the surface of the support. It should be noted that all hybrids obtained following modification were washed in propan-2-ol with the use of an ultrasound bath (Polsonic, Poznan, Poland) for 30 min to remove all unbound POSS particles.

The SEM images indicate that the proposed washing procedure has no influence on the structure of the composite-POSS particles are present on the chitinous substrate. This suggests that POSS particles are tightly bonded to the chitin surface.

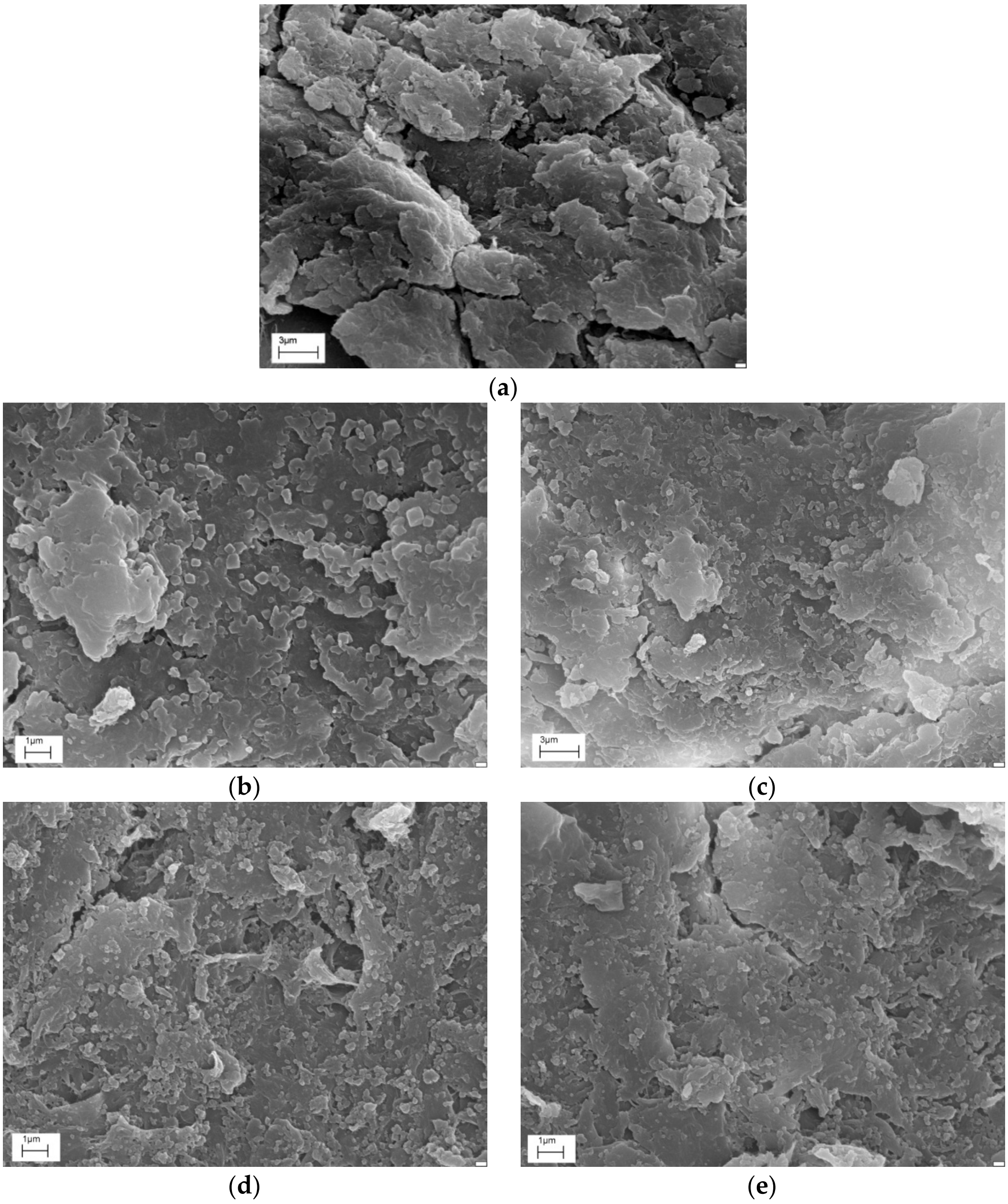

Figure S1. Scanning electron microscopy (SEM) images of (**a**) α-chitin and obtained hybrid materials: (**b**) chitin-APOSS; (**c**) chitin-EPOSS; (**d**) chitin-VPOSS; and (**e**) chitin-MPOSS.

2.1.2. Fourier Transform Infrared Spectroscopy (FTIR) Results

The Fourier transform infrared spectroscopy (FTIR) spectra (Figure S2) present the results of analysis of pure chitin, the modifiers used and the hybrid materials produced following functionalization using POSS compounds in concentrations of 2.5 and 5.0 g. The SEM images indicate that the proposed washing procedure has no influence on the structure of the composite-POSS particles are present on the chitinous substrate. This suggests that POSS particles are tightly bonded to the chitin surface.

The spectra of the products indicate the effective functionalization of the chitin surface with compounds in the oligosilsesquioxanes group. Evidence for this is provided by the characteristic signals appearing on the spectra of all of the obtained systems at wavenumber 1100 cm^{-1}, originating from stretching vibrations of Si–O bonds, as well as three bands between 1000 and 470 cm^{-1} produced by symmetric and asymmetric stretching and bending vibrations of Si–O–Si. Compared with the chitin spectrum, the spectra of the resulting hybrids have markedly more intense signals between 2950 and 2850 cm^{-1}, generated by stretching vibrations of C–H bonds. Further confirmation

of the effective modification of the surface is provided by bands in the wavenumber range 1400–1200 cm⁻¹, produced by stretching vibrations of Si–C bonds and appearing on the spectra of all of the systems obtained. In addition, in the case of each of the hybrid products, there is an intense signal at 1487 cm⁻¹ attributable to bending vibrations of C–H bonds.

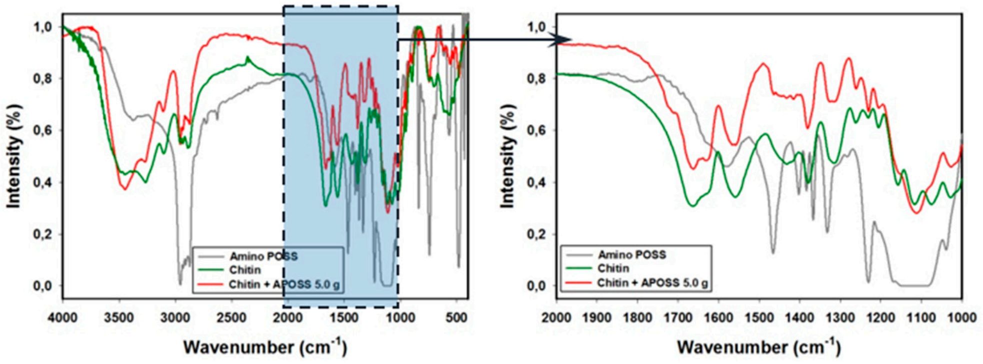

(**a**)

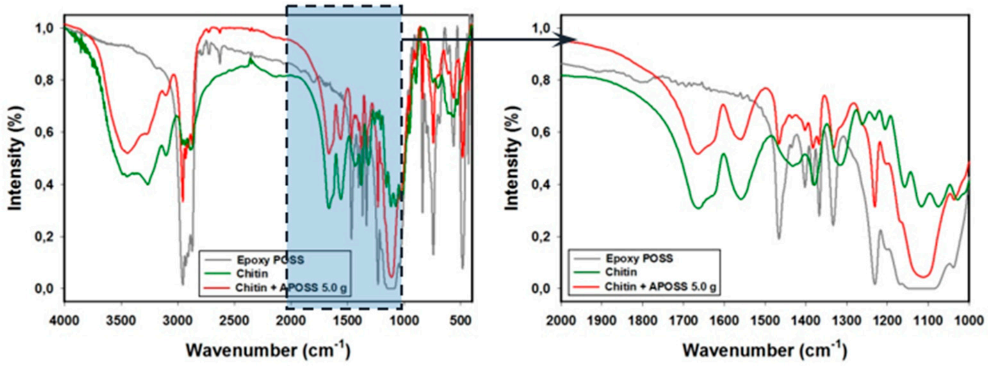

(**b**)

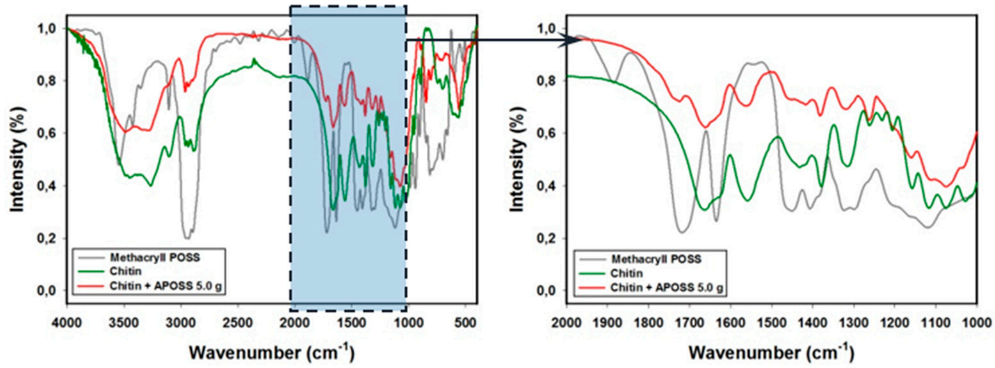

(**c**)

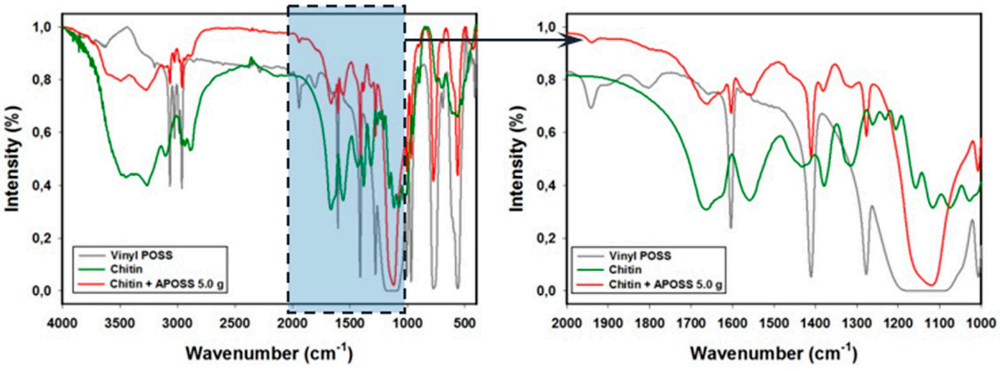

(**d**)

Figure S2. FTIR spectra of α-chitin, POSS compounds and the resulting hybrid materials: (**a**) chitin-APOSS; (**b**) chitin-EPOSS; (**c**) chitin-VPOSS; (**d**) chitin-MPOSS.

In the case of the chitin-APOSS hybrid, particular attention should be paid to the signal with a maximum at wavenumber 3216 cm^{-1}, appearing as a result of stretching vibrations of N–H bonds, and the signal at 1583 cm^{-1}, associated with deformation vibrations of the same groups.

On the spectra of the products formed by functionalization of the chitin surface with VPOSS, the most significant signals are those at wavenumbers 3065 and 3024 cm^{-1}, resulting from symmetric stretching vibrations of =C–H bonds, and that at 1945 cm^{-1}, related to the presence in the structure of C=C double bonds [2].

Confirmation of the effective modification of chitin with MPOSS is provided by the signals at 3102 cm^{-1}, resulting from stretching vibrations of =C–H bonds, and at 1893 cm^{-1}, generated by C=C bonds. The spectra of the products also feature signals at 1723 and 1447 cm^{-1}, originating, respectively, from vibrations of C=O and C–O, present in the ester bond. It should be noted that a significant factor affecting the modification of the chitin surface is the quantity of POSS compound used. The spectra of all materials obtained using 5.0 g of POSS contain more intense signals, indicating that the support has been functionalized with greater quantities of the modifier.

2.1.3. ^{13}C CP MAS NMR Analysis

Figure S3 shows the ^{13}C CP MAS NMR spectra of the chitin, the polyhedral oligomeric silsesquioxanes and the obtained hybrid materials: (a) chitin + EPOSS 5.0 g and (b) chitin + VPOSS 5.0 g.

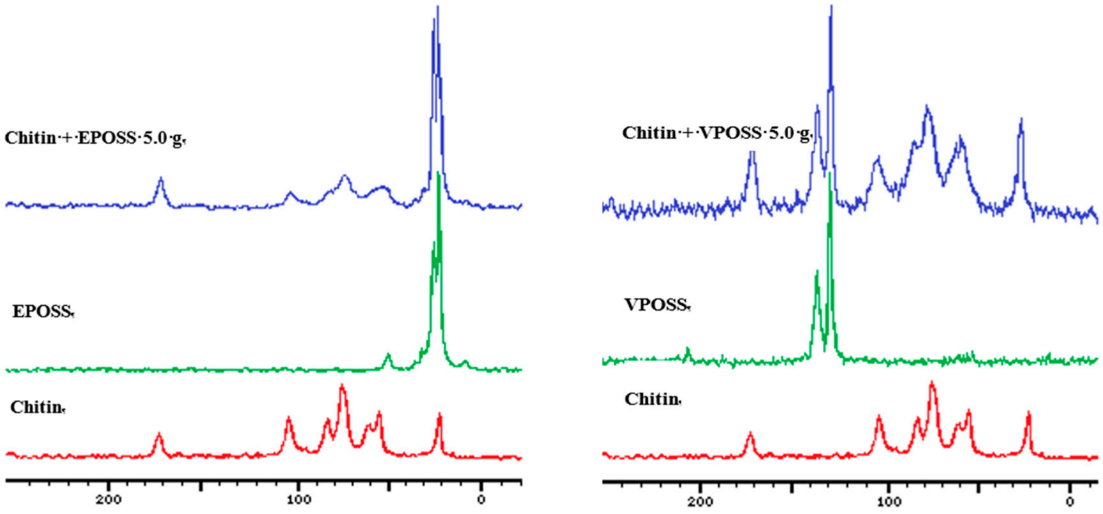

(**a**) (**b**)

Figure S3. ^{13}C CP MAS NMR spectra of α-chitin, POSS compounds, and (**a**) chitin + EPOSS 5.0 g and (**b**) chitin + VPOSS 5.0 g.

The signals appearing on the spectrum of the commercial product used for modification are characteristic for α-chitin [3,4] while the bands appearing on the spectra of the POSS materials used are in accordance with the functional groups contained in the structure of those compounds [5,6]. The spectra of the products contain signals generated both by the support and by the modifier used. Attention should be drawn to the signal at 20.6 ppm on the spectrum for the chitin + EPOSS 5.0 g sample, which is more intense in the modified material, in view of the similar chemical environment of the CH_3 group in chitin and CH_2 in the applied POSS. A detailed description of the signals generated by the particular groups and the values of their chemical shifts are given in the Table S1.

The results of ^{13}C CP MAS NMR analysis confirm the obtaining of hybrid materials on the basis of chitin and the selected POSS compounds.

Table S1. The chemical shift value (δ, ppm) of 13C CP MAS NMR spectrum of chitin, EPOSS, VPOSS, and obtained hybrid materials.

Chitin	EPOSS	VPOSS	Chitin + EPOSS 5.0 g	Chitin + VPOSS 5.0 g	Assignment
–	8.7	–	8.9	–	Si–C^1–C
20.8	–	–	20.6	21.7	CH_3 acetamide groups
–	21.1	–		–	Si–C–C^2
–	21.6	–	21.0	–	C5 cyclohexane ring
–	27.4	–	27.0	–	C2 cyclohexane ring
–	31.3	–	31.9	–	C1 cyclohexane ring
–	44.7	–	45.0	–	C3, C4 cyclohexane ring with epoxy group
–	45.3	–		–	
51.6	–	–	51.1	52.0	C2 hexose ring
55.4	–	–	53.2	55.6	C6 hexose ring
74.2	–	–	73.4	74.3	C3 hexose ring
75.6	–	–	74.7	74.4	C5 hexose ring
84.9	–	–	83.2	83.6	C4 hexose ring
102.1	–	–	101.4	98.7	C1 hexose ring
–	–	125.7	–	125.5	Si–C=C^2
–	–	133.8	–	133.7	Si–C^1=C
175.8	–	–	175.4	176.8	C=O acetamide groups

2.1.4. Raman Spectroscopy Results

Table S2. Raman shifts (cm^{-1}) and the proposed assignments of the chitin, POSS compounds, and obtained hybrid materials.

Chitin	Hybrid Materials Chitin-				Assignments	Oligomeric Polihedral Silsesquioxanes			
	E POSS	A POSS	M POSS	V POSS		E POSS	A POSS	M POSS	V POSS
–	403<br>463	403<br>463	–	–	out of plane bending Si–O–Si	410<br>464	410<br>464	–	–
–	–	–	628	–	in plane bending O–C=O	–	–	630	–
–	843	–	–	–	δ C–Si–C	849	849	850	–
–	1038	–	–	–	δ C–C, Si–O–Si stretching	1043	1043	1006	1013
–	1230	–	–	–	wagging δ CH_2	1230	1230	–	–
1268	1262	–	1265	1278	Amide III/ twisting δ CH_2	1268	1268	1260	1277
1418	1403	1399	1415	1412	δ CH_2/ scissoring δ CH_3	1407	1407	1404	1414
1452	1448	1444	1454	–	twisting δ CH_3	1449	1449	1455	–
–	1460	1467	–	–	rocking δ CH_3	1462	1462	–	–
–	–	1508	–	–	deformation N–H	–	1499	–	–
–	–	–	–	1605	C=CH_2 (vinyl) stretching	–	–	–	1602
–	–	–	1640	–	stretching C=O with hydrogen bond	–	–	1638	–
–	–	–	1720	–	C=O (ester) stretching	–	–	1715	–
–	2718	2718	–	–	CH stretching	2716	2716	–	–
2883	2869	2869	2882	2879	CH_2 stretching	2869	2869	–	–
-	-	-	-	2991, 3066	stretching CH vinyl	-	-	-	2961, 3062

References

1. Wang, B.; Li, J.; Zhang, J.; Li, H.; Chen, P.; Gu, Q.; Wang, Z. Thermo-mechanical properties of the composite made of poly (3-hydroxybutyrate-*co*-3-hydroxyvalerate) and acetylated chitin nanocrystals. *Carbohydr. Polym.* **2013**, *95*, 100–106.
2. Chen, J.; Hanomn, M.A.; Hu, H.; Chen, Y.; Rao, A.M.; Eklund, C.P.; Haddon, R.C. Solution properties of single-walled carbon nanotubes. *Science* **1998**, *282*, 95–98.
3. Cardenas, G.; Cabrera, G.; Taboada, E.; Miranda, S.P. Chitin characterization by SEM, FTIR, XRD, and ^{13}C cross polarization/mass angle spinning NMR. *J. Appl. Polym. Sci.* **2014**, *93*, 1876–1885.
4. Spinde, K.; Kammer, M.; Freyer, K.; Ehrlich, H.; Vournakis, J.N.; Brunner, E. Biomimetic silicification of fibrous chitin from diatoms. *Chem. Mater.* **2011**, *23*, 2973–2978.
5. Strachota, A.; Whelan, P.; Kriz, J.; Brus, J.; Urbanova, M.; Slouf, M.; Matejka, L. Formation of nanostructured epoxy networks containing polyhedral oligomeric silsesquioxane (POSS) blocks. *Polymer* **2007**, *48*, 3041–3058.
6. Zheng, L.; Kasi, R.M.; Farris, R.J.; Coughlin, E.B. Synthesis and thermal properties of hybrid copolymers of syndiotactic polystyrene and polyhedral oligomeric silsesquioxane. *J. Polym. Sci. Part A* **2002**, *40*, 885–891.
